# Total marrow irradiation in hematopoietic stem cell transplantation for hematologic malignancies

**DOI:** 10.3389/fmed.2023.1155954

**Published:** 2023-04-20

**Authors:** Mariana Nassif Kerbauy, Leonardo Javier Arcuri, Sergio Leonardo Favareto, Ana Carolina Pires de Rezende, Nelson Hamerschlak

**Affiliations:** ^1^Department of Hematology and Bone Marrow Transplantation, Hospital Israelita Albert Einstein, São Paulo, Brazil; ^2^Academic Research Organization, Hospital Israelita Albert Einstein, São Paulo, Brazil; ^3^Department of Radiation Oncology, Hospital Israelita Albert Einstein, São Paulo, Brazil

**Keywords:** hematopoietic stem cell transplant, bone marrow transplantation, total body irradiation, total marrow irradiation, total marrow and lymphoid irradiation, targeted marrow irradiation, conditioning regimen, multiple myeloma

## Abstract

Total body irradiation (TBI) has been an essential component of the conditioning regimen in hematopoietic cell transplantation for many years. However, higher doses of TBI reduce disease relapse at the expense of more significant toxicities. Therefore, total marrow irradiation and total marrow and lymphoid irradiation have been developed to deliver organ-sparing targeted radiotherapy. Data from different studies show that TMI and TMLI can be safely administered in escalating doses in association with different chemotherapy conditioning regimen protocols, in situations with unmet needs, such as multiple myeloma, high-risk hematologic malignancies, relapsed or refractory leukemias, and elderly or frail patients, with low rates of transplant-related mortality. We reviewed the literature on applying TMI and TMLI techniques in autologous and allogeneic hematopoietic stem cell transplantation in different clinical situations.

## Introduction

Total body irradiation (TBI) has been an essential component of the conditioning regimen in hematopoietic cell transplantation (HCT) for many years. In patients with malignant diseases, the conditioning regimen has two main objectives: (1) to reduce tumor burden and disease relapse after transplant and (2) to provide sufficient immunoablation to prevent rejection. TBI has been widely used due to its immunosuppressive profile and effectiveness against leukemias and lymphomas, even in sanctuary sites such as the brain and testes (1). Higher doses of TBI reduce disease relapse at the expense of more significant toxicities, especially in the lungs, liver, bowel, thyroid, and gonads. It also leads to impaired growth and development in children, fertility issues, secondary malignancies, and increased transplant-related deaths (2). To decrease organ toxicity, fractionated TBI was an effective strategy due to a higher proportion of intact repair mechanisms retained in normal tissues as opposed to cancer cells (3), and hyperfractionated TBI and partial lung shielding have reduced fatal interstitial pneumonitis from 50% in the single-dose regimen to 18%, with 100% engraftment rate (4).

The need for reducing TBI toxicity to critical organs while increasing doses to selected targets through technical optimization has emerged as an essential need for the bone marrow transplant team and radiation oncologists. Total marrow irradiation (TMI) and total marrow and lymphoid irradiation (TMLI) were developed to deliver organ-sparing targeted radiotherapy using intensity-modulated radiation therapy (IMRT). The term TMI is usually used if the target is the bone and bone marrow, and TMLI adds the spleen and major lymph node chain. As a result, radiation to structures such as the lenses, oral cavity, thyroid, parotid glands, esophagus, lungs, heart, liver, stomach, kidneys, urinary bladder, genitalia, and small bowel are usually minimized.

Total marrow irradiation was usually used in autologous HCT for multiple myeloma and TMLI in allogeneic HCT (5). Data from different studies show that TMI and TMLI can be safely delivered at an escalated dose in association with different chemotherapy conditioning regimen protocols. Our objective is to review literature data regarding conditioning regimes and the application of TMI techniques in autologous and allogeneic TMI. Thus, we performed a search in PubMed with the following strategy: (“Total marrow”[Title]) OR (“Targeted marrow”[Title]) OR (“Targeted-marrow”[Title]) OR (TMI[Title] AND transplant^*^[Title]).

## TMI treatment planning

The main targets for TMI planning purposes are bones and bone marrow. For TMLI, the spleen and major lymph node chains are also included. Depending on the institution's protocol, the liver, brain, and testes may also be included. There are several protocols with different radiation dose prescriptions, ranging from 6 to 20 Gy (5, 6). The irradiation protocol starts with the simulation, outline of targets and organs at risk (OAR), planning (dose calculation) and, finally, the delivery of the treatment.

A computed tomography (CT) scan with 5–10 mm is performed for the simulation. Patients usually stay in the supine position, with immobilization devices such as vacuum bags and/or thermoplastic masks. In addition, 4D CT scans of the chest and abdomen may be utilized to account for any organ movement during respiration (5).

The contouring begins with defining the target volumes and the avoidance structures. First, the clinical target volume (CTV) is defined as the spleen and entire bony skeleton, excluding the mandible. After that, anisotropic margins to account for possible uncertainties in beam alignment, patient positioning, organ motion, and organ deformations are generated to create the planning target volume (PTV). Avoidance structures, also known as organs at risk, include lenses, parotid glands, oral cavity, thyroid, esophagus, lungs, heart, liver, stomach, kidneys, small bowel, urinary bladder, and genitalia (6).

The treatment may be delivered by a regular linear accelerator (LINAC) or TomoTherapy^®^ (combines intensity-modulated radiation therapy (IMRT) with a spiral delivery pattern). For LINAC, the planning is generated using 4–5 isocentric volumetric modulated arc therapy techniques to the upper body. The lower extremities are planned and treated with junctioned AP-PA fields (7, 8). Volumetric imaging (image-guided radiation therapy – IGRT) is used for daily patient setup (5).

## TMI in autologous HCT

Total marrow irradiation in autologous conditioning regimen for multiple myeloma was pioneered by Einsele et al. (9). Eighty-nine patients with stage II–III multiple myeloma, *de novo* or pretreated, received TMI 9 Gy, 12 mg/kg oral busulfan (equivalent to 9.6 mg/kg intravenous busulfan), and cyclophosphamide 120 mg/kg in patients. Three patients had sinusoidal obstruction syndrome (SOS). Transplant-related mortality was as low as 2%. Median progression-free survival was 36 months for patients with *de novo* MM. Complete response increased from 2% to 44% following chemoradiotherapy (Table 1). In the current days, the use of intravenous busulfan may attenuate the incidence of SOS.

**Table 1:** Clinical results of TMI in autologous transplants for multiple myeloma.

**Author, year**	**Study type**	***N***	**Drugs**	**TMI dose**	**% Relapsed disease**	**Complete response**	**VGPR**
Cailleteau et al. (10)	Phase I	13	Melphalan 140 mg/m2	Max 14 Gy	100%	31%	69%
Ladbury et al. (11)	Phase I/II	54	-	MTD 16 Gy	0%	48%	70%
Giebel et al. (12)	Phase II	50	-	12 Gy	0%	42%	NA
Patel et al. (13)	Phase I	12	Melphalan 200 mg/m2	Max 9 Gy	100%	NA	73%
Lin et al. (14)	Randomized	3	Melphalan 140 mg/m2	8 Gy	0%	-	-
Somlo et al. (15)	Phase I	25	-	MTD 16 Gy	0%	23%	73%
Einsele et al. (9)	Phase I/II	89	Busulfan 12 mg/kg Cyclophosphamide 120 mg/kg	9 Gy	0%	44%	56%

TMI, total marrow irradiation; VGPR, very good partial response; MTD, maximum tolerated dose.

Cailleteau et al. (10) reported the results of a phase I trial of melphalan 140 mg/m2 combined with TMI before autologous HCT for patients in their first relapse. Thirteen patients were included. Four dose levels were explored: 8 Gy, 10 Gy, 12 Gy, and 14 Gy. The dose administered to the lungs was systematically below 8 Gy. Maximum tolerated dose was not reached, and the rate of acute toxicity was low. Pre-TMI rate of CR was 15% and the post-TMI rate of CR was 31%, showing that TMI increased the depth of response in these patients without increasing toxicity. Patel et al. (13) combined up to 9 Gy of TMI with melphalan 200 mg/m^2^ in 12 patients with relapsed myeloma. The maximum tolerated dose was not achieved, there was no grade 4 non-hematologic toxicity, and median overall survival was around 3 years. Although feasible, maximal doses of TMI and melphalan, when combined, have yet to be established.

Ladbury et al. (11) reported the results of a unicentric protocol that included a second autologous HCT with single-agent TMI (doses: 10–18 Gy) after a first transplant based on melphalan 200 mg/m^2^. Thirty patients received the second HCT based on TMI. The maximum tolerated dose was 16 Gy. In a long follow-up (12.3 years among survivors), the progression-free survival at 10 years was 20%. There were five cases of secondary malignancies and an additional five cases of non-melanoma skin cancers. Somlo et al. (15) also tested a second high-dose therapy based on TMI (doses: 10–18 cGy) in 22 patients. The maximum tolerated dose was also 16 Gy. In a quite similar strategy, Giebel et al. (12) published the results of a tandem HCT strategy with 12 Gy in the first course and melphalan 200 mg/m^2^ in the second one. Fifty patients were included. Five-year progression-free survival was 55%. The authors showed the anti-myeloma activity of TMI monotherapy since VGPR increased from 46% to 74% after the first transplant and 86% after the second transplant. The rate of complete response changed from 10% before the first auto-HCT to 42% after tandem transplantation. Non-hematological complications were infrequent and only 14% of patients had mucositis grades 2–4. Both studies have shown that the maximal dose of TMI, when used as a single agent, is 16 Gy.

Lin et al. (14) tried to compare the results of autologous HCT for multiple myeloma with TMI 8 Gy combined with melphalan 140 mg/m2 with single-agent melphalan 200 mg/m2, but the small number of patients hampered further analyses.

Most of these reports do not represent the current practice in multiple myeloma, and these strategies should be tested in the context of highly active induction regimens and maintenance therapy.

## TMI in matched-related and unrelated HCT

The safety and efficacy of TMI and TMLI as part of the conditioning regimen in allogeneic HCT have been evaluated in different publications over the years (Table 2).

**Table 2:** Clinical results of TMI/TMLI in matched-donor allogeneic transplants.

**Author, year**	**Type of study**	***N***	**Disease**	**Disease status at transplant**	**Conditioning regimen**	**TMI dose**	**Lymphoid irradiation**	**TRM**	**OS**	**Relapse**
Ogawa et al. (16)	Prospective phase I trial	9	ALL and CMML	Complete response	Cyclophosphamide and TMLI	14–18Gy	Yes	0	1 y:83%	33,3% (3 cases)
Ali et al. (6)	Prospective phase I trial	23	Hematological malignancies	73% had active or measurable residual disease at transplant	Busulfan (2 days) and fludarabine	6Gy	No	1y:4%	1y 65%	1y: 43%
Stein et al. (17)	Prospective phase II trial	18	AML	Complete response and (MRD)-negative	TMLI and PTCy	20Gy	Yes	2y:0%	2y:86.7%	2y:16.7%
Haraldsson et al. (18)	Prospective observational study	37	Hematological malignancies	Complete and partial response	TMI-based	12 Gy	No	-	1y GRFS: 67.5%	-
Shi et al. (19)	Retrospective study	61	Hematological malignancies	20/ patients with refractory leukemia	TMI/ TMLI hypo-fractionation	10 Gy	Yes	2 y:5%	2 y:74.7%	27%
Jensen et al. (20)	Prospective study	61	Hematological malignancies	Complete and partial response and active disease	Fludarabine, Melphalan, TMLI	12 Gy	Yes	5 y:41%	5 y:33%	5 y:26%
Stein et al. (21)	Prospective phase I study	51	Acute leukemia	relapsed/refractory	Cyclophosphamide and etoposide, TMLI	12–20 Gy	Yes	1y:3.9%	1y:55.5%	64%
Hui et al. (22)	Prospective phase I study	12	Acute leukemia and prolymphocytic leukemia	Complete and partial response and active disease	Cyclophosphamide and fludarabine, TMI	15 Gy and 18 Gy	No	1 y:42%	1 Y:42%	1 y:36%
Patel et al. (23)	Prospective phase I study	14	High-risk hematologic malignancies	Complete response and active disease	Busulfan and fludarabine, TMI	3–12 Gy	No	3 y:28%	3 y:50%	
Rosenthal et al. (24)	Prospective Phase 1/2 study	33	Advanced hematologic disease	Complete and partial response and active disease	Fludarabine, Melphalan, TMLI	12 Gy	Yes	2 y:25%	1 y:75%	21%
Wong et al. (25)	Prospective phase I study	12	Advanced AML/ALL	Induction failure and first relapse	TMI, etoposide and cyclophosphamide	12–15 Gy	No	16%	50%	33.3%
Wong et al. (25)	Prospective phase I study	20	Advanced AML/ALL	Induction failure, first and second relapse	Busulfan and etoposide, TMI	12–13.5Gy	No	40%	25%	35%

ALL, acute lymphoblastic leukemia; AML, acute myeloid leukemia; CMML, chronic myelomonocytic leukemia; TMLI, total marrow and lymphoid irradiation; TMI, total marrow irradiation; TRM, transplant-related mortality; OS, overall survival; PTCy, post-transplant cyclophosphamide; GRFS, graft-vs.-host disease-relapse-free survival.

Ali et al. (6) evaluated RIC conditioning with busulfan (2 days) and fludarabine with TMI 6 Gy in 26 patients with high-risk hematologic malignancies not eligible for myeloablative transplantation. In this publication, the median age was 64 years, and 73% had active or measurable residual disease at transplantation. They included 18 matched-unrelated donors, five matched sibling donors, and three haploidentical donors. All patients engrafted neutrophils. The 1-year overall survival was 65%, the 1-year TRM was 4%, and the 1-year cumulative incidence of relapse was 43%, demonstrating a feasible intensification of RIC conditioning with TMI in medically frail patients with high-risk disease. These results show that low-dose TMI combined with a RIC conditioning regimen leads to a high engraftment rate. The relapse rate was relatively high, although the frequency of active disease prior to HCT was also high.

Reduced-intensity conditioning (RIC) may provide reasonable disease control in frail patients with high-risk hematologic malignancies undergoing allogeneic HCT. Wong et al. (28), Rosenthal et al. (24), and Jensen et al. (20) prospectively studied the association of a RIC conditioning regimen with melphalan and fludarabine associated with TMLI 12 Gy. Wong et al. evaluated toxicities in eight patients with hematologic malignancies with this conditioning regimen, and grades 2–3 nausea, vomiting, mucositis, and diarrhea were observed, with no grade 4 non-hematologic toxicity. Rosenthal et al. (24) included 33 patients over 50 years or with compromised organ function and showed the median time to neutrophil engraftment of 14 days, 1-year OS of 75%, 2-year NRM of 25%, and 21% deaths due to progressive disease. Jensen et al. evaluated the clinical outcomes of 61 patients with a median age of 55 years and 5-year OS, NRM, and relapse were 44%, 33%, and 26%, respectively (20).

Ogawa et al. (16) tested myeloablative conditioning consisting of cyclophosphamide 60 mg/kg/day for 2 days and TMLI with doses of 14, 16, and 18 Gy for 3 days in 3+3 design in nine patients with acute lymphoblastic leukemia or chronic myeloblastic leukemia with unrelated donors. All patients achieved neutrophil engraftment at a median of 19 days. No patient showed dose-limiting toxicities, and 1-year overall survival was 83.3%. There were three disease relapses and no documented TRM. Stein et al. (21) evaluated TMLI with a dose ranging from 12 to 20 Gy in 51 patients with active relapsed/refractory leukemia undergoing HCT with matched-related or unrelated donors, combined with cyclophosphamide and etoposide. The maximum tolerated dose was 20 Gy. All patients had neutrophil engraftment at a median of 15 days. In this high-risk population, 88% of patients achieved complete response at D+30. With a median follow-up of 24.6 months, 33 patients relapsed. The 1-year OS was 55.5%, and the 1-year TRM was 3.9%. Hui et al. (22) evaluated TMI in a phase I dose-escalation trial with 12 patients who received conditioning therapy with cyclophosphamide and fludarabine in conjunction with TMI at 15 Gy and 18 Gy (in 3 Gy/fractions) while maintaining TBI dose to vital organs at < 13.2 Gy. The median time for neutrophil recovery was 26 days. The 1-year OS was 42%, 1-year relapse was 36%, and 1-year TRM was 42%. Although excessive specific organ toxicity was not found, the authors decided to suspend enrollment in the 18 Gy arm due to 50% transplant-related mortality. In summary, these studies show that higher doses of TMI combined with cyclophosphamide-based chemotherapy are feasible and that the maximum tolerated dose of TMI might not have been reached.

In a phase I trial published by Patel et al. (23), TMI 3 Gy, 6 Gy, 9 Gy, and 12 Gy were combined with myeloablative fludarabine and busulfan in 14 patients with high-risk hematologic disease undergoing HCT with HLA-matched related, unrelated, or mismatched donor using peripheral blood grafts. All patients engrafted promptly at a median of 15 days. Extrahematologic toxicities were limited to grades 1–2. With a median follow-up of 3 years, the OS was 50%, with four deaths caused by transplantation-related complications and three due to relapse.

Wong et al. (25) presented 2 phase I trials of a combination of TMI with higher-intensity chemotherapy in patients with advanced leukemias. The first trial consisted of a TMI dose of 12–15 Gy combined with etoposide (60 mg/kg) and cyclophosphamide (100 mg/Kg). The median age of the 12 included patients was 33 years, with a median follow-up of 14.7 months, and five patients remained in CR, and no dose-limiting toxicity was achieved. The second trial consisted of TMI 12–15.5 Gy associated with busulfan (4 days) and etoposide (30 mg/kg). They included 20 patients with a median age of 41 years. Grade 4 dose-limiting toxicities were seen at 13.5 Gy (stomatitis and hepatotoxicity) (25). Therefore, doses of TMI combined with higher-intensity, busulfan-based chemotherapy, probably should be limited to 12 Gy.

Haraldsson et al. (18) compared 37 patients who underwent allogeneic transplants and received TMI to a retrospective control of 33 patients that had received TBI. Dose distribution for TMI patients was kept to a minimum in kidneys, heart, bowel bag, and liver. The 1-year GVHD-free, relapse-free survival (GRFS) was 67.5% for TMI and 39.4% for TBI (*p* = 0.03), and this difference was higher in patients receiving MUD HCT (*p* < 0.01). No significant differences in neutrophil recovery, overall survival (OS), transplant-related mortality (TRM), or relapse were found. Therefore, compared with TBI, TMI may offer superior results in terms of GRFS. A recent study compared dosimetric changes in irradiation received by the target volume and organs at risk between TBI and TMI plans. The authors theoretically calculated TMI plans for 35 patients who had already received TBI conditioning. In this analysis, TMI significantly reduced the dose in organs at risk, with mean dose reduction in the liver by 49% and spleen by 55–59%, and achieved the prescribed dose in the target volume (29).

Shi et al. (19) evaluated 61 patients with hematologic malignancies who underwent HLA-matched related or unrelated HCT with peripheral blood stem cells. In eight patients, the conditioning regimen consisted of TMI, and in 53 patients, TMLI. Patients received 8 Gy in the bone marrow in a single day treatment and 10 Gy in the involved field in two fractions a day associated with GVHD prophylaxis with tacrolimus and sirolimus. None of the patients had grades 3–4 non-hematologic toxicities. The 2-year OS was 74.7%, 2-year TRM was 5%, and the relapse rate was 27%, demonstrating that the hypo-fractionation TMI/TMLI scheme may be an alternative in this scenario.

Graft-vs.-host disease (GVHD) is a significant cause of morbidity and mortality after allogeneic stem cell transplantation, and comparisons between haploidentical HCT with post-transplant cyclophosphamide (PTCy) and unrelated donor (URD) HCT have shown comparable overall survival with lower incidences GVHD with PTCy. Therefore, in recent years, PTCy has been expanded to matched transplants. Stein et al. (17) have recently published a study testing TMLI at 20 Gy with PTCy for patients with acute myeloid leukemia in the first or second complete response undergoing matched donor allogeneic HCT. The patient safety lead-in segment followed a 3+3 dose expansion cohort of up to 12 additional patients. PTCy was administered 50 mg/kg on days +3 and +4 after infusion, and tacrolimus was given until day +90 and then tapered. All patients were engrafted. The authors demonstrated the feasibility of a chemotherapy-free conditioning regimen with a 2-year OS of 86.7%, 2-year TRM of 0%, 2-year relapse of 16.7%, and GRFS rate of 59.3% after a median follow-up of 24.5 months.

Another recent study evaluated 50 patients with hematologic malignancies who underwent conditioning with fludarabine, thiotepa, cyclophosphamide, and TMLI total dose of 13.5 Gy and TLI of 11.5 Gy. They included 11 matched-related donors and 39 haploidentical donors. Patients received donor regulatory T cells and conventional T cells before infusion, followed by CD34+ selected grafts on day 0, with no pharmaceutical immunosuppressive therapy. Eighteen patients (36%) had grade II acute GVHD, and the mean TMLI dose to the whole intestine was 7.1 Gy (30).

Extramedullary (EM) relapse in patients receiving TMI or TMLI is a concern in allogeneic HCT. Kim et al. (31) evaluated 101 patients enrolled in TMLI trials between 2006 and 2012, with total radiation doses ranging from 12 to 15 Gy. In this population, EM relapse occurred in 13 patients (12.9%) and bone marrow relapse in 25.7%, comparable to published results with regimens including TBI. Of the 13 patients, seven had the extramedullary disease before HCT, which was the only significant predictor of subsequent EM disease (31).

As TMI decreases radiation in organs at risk and allows dosage escalation to improve oncologic outcomes, subsequent malignant neoplasms (SMNs) are another concern. Han et al. (32) retrospectively compared the estimated radiation-induced, organ-specific, secondary solid-tumor rates in 20 patients who received TMI (10 patients received 12 Gy and 10 patients 20 Gy with 12 Gy to the brain and liver) to a generated conventional TBI treatment plan with a prescription dose of 12 Gy and showed TMI could significantly reduce overall radiation-induced secondary solid-tumor. Another recent publication compared SMN in patients submitted to TBI (171 patients) or TMI-based conditioning regimens (171 patients) to a 12–20 Gy dose. TMI patients received higher radiation doses (16 Gy vs. 13.2 Gy), with no significant difference in the risk of SMN in the two cohorts (nine patients in the TBI group and 10 in the TMI group), and no patients developed a subsequent hematologic malignancy (33).

## TMI in haploidentical HCT

Using the PTCy platform has revolutionized haploidentical HCT with low rates of GVHD and TRM. However, relapse remains a concern in high-risk patients. In this context, TMI and TMLI may be an alternative to reduce toxicities without hampering disease control (Table 3). A recent phase I trial has been published, including 31 patients with high-risk leukemias or myelodysplastic syndrome. The conditioning regimen was based on TMLI with increasing doses from 12 to 20 cGy associated with fludarabine and cyclophosphamide. PTCy with tacrolimus and mycophenolate mofetil was the GVHD prophylaxis. All patients achieved neutrophil recovery at a median of 17 days. In addition, the authors demonstrated a 2-year NRM of 13%, a cumulative incidence of grade II to IV acute GVHD at day 100 of 52%, and acute GVHD grades III to IV of 6%. In the group of patients who received recommended TMLI dose at 20 Gy, 1-year overall survival was 83% and 1-year relapse of 17% (26), demonstrating the feasibility and safety of TMLI combined with PTCy.

**Table 3:** Clinical results of TMI/TMLI in haploidentical allogenic transplants.

**Author, year**	**Type of study**	***N***	**Disease**	**Disease Status at transplante**	**Conditioning regimen**	**TMI dose**	**Lymphoid irradiation**	**TRM**	**OS**	**Relapse**
Al Malki et al. (26)	Prospective phase I study	31	leukemias or myelodysplastic syndrome	Complete and partial response and active disease	Fludarabine, cyclophosphamide and TMLI and PTCy	12–20Gy	Yes	2y:13%	1y:83% (20Gy)	1y:17% (20Gy)
Ali et al. (6)^*^	Prospective phase I trial	3	Hematological malignancies	73% had active or measurable residual disease at transplant	Busulfan (2 days) and fludarabine, TMI and PTCy	6Gy	No	1y:4%	1y 65%	1y: 43%
Sarina et al. (27)	Retrospective study	59	HL, NHL, CLL/PLL	Complete and partial response and active disease	Fludarabine, cyclophosphamide and TMLI and PTCy	2Gy	Yes	2y:23%	2y:63%	2y:23%

^*^Data of haploidentical were not presented separately.

TMLI, total marrow and lymphoid irradiation; TMI, total marrow irradiation; TRM, transplant-related mortality; OS, overall survival; PTCy, Post-transplant cyclophosphamide; HL, Hodgkin lymphoma; NHL, non-Hodgkin lymphoma; CLL, chronic lymphocytic leukemia; PLL, prolymphocytic leukemia.

Sarina et al. (27) evaluated TMLI 2 Gy instead of TBI 2 Gy associated with a non-myeloablative (NMA) conditioning regimen with cyclophosphamide, fludarabine in patients who underwent haploidentical transplantation with PTCy, calcineurin inhibitor, and mycophenolate mofetil. Fifty-nine patients were included in TMLI and 37 in the TBI group, and, in multivariable analysis, TMLI did not influence OS, TRM, or PFS compared with TBI. This finding is relatively expected since TBI 2 Gy is seldom toxic.

Ali et al. (6) have included three haploidentical transplants in the cohort of patients with high-risk hematologic malignancies submitted to RIC Bu/Flu conditioning associated with TMI. These patients received tacrolimus, MMF, and PTCy, and the authors showed the feasibility of this association in frail patients, although the low number of patients hampers further conclusions.

In summary, these studies show that TMLI and TMI in doses up to 20 Gy are feasible with PTCy and RIC or NMA conditioning regimens. To the best of our knowledge, there is no data on TMLI or TMI combined with myeloablative doses of chemotherapy and PTCy.

## Discussion

The data reviewed herein show that TMI and TMLI are feasible and safe and may reduce TBI toxicities associated with different chemotherapy protocols. Furthermore, it can be delivered to other populations, but only a few prospective trials have been published so far, with small samples restricted to phase I or II clinical trials.

In the autologous HCT scenario, some trials with multiple myeloma patients have been done. Multiple myeloma is caused by the abnormal proliferation of plasma cells and accounts for about 15% of all hematological malignancies (34). Over the last 20 years, autologous HCT has been considered the standard therapy for transplant-eligible patients with multiple myeloma, as it improves progression-free survival compared to schemes without HCT, even with new therapies in recent years (35, 36). The most frequently used conditioning regimen is melphalan 200 mg/m^2^, but different schemes have been evaluated to increase response rates and progression-free survival. Since multiple myeloma is highly sensitive to radiotherapy, some studies have evaluated the combination of chemotherapy with total body irradiation (TBI), but with no benefit in overall survival because of increased toxicities (37, 38). In this scenario, TMI was evaluated in association with different chemotherapy protocols or as a second HCT and showed that high doses of TMI may be delivered upfront or in the relapse setting. However, as the treatment of multiple myeloma has been revolutionized with new treatments in recent years, these strategies should be tested in the context of highly active induction and maintenance regimens.

In the allogeneic HCT scenario, higher intensities of the conditioning regimens reduce the chance of relapse of the underlying disease (39), at the expense of increased transplant-related mortality, including regimens with TBI (40).

For elderly patients or patients with comorbidities, different reduced-intensity conditioning protocols have been increasingly used, and a conditioning regimen that changes the balance point with lower transplant-related mortality and higher cure rates is desirable. Therefore, TMI and TMLI may be an alternative to these frailer patients. Their feasibility and safety have been demonstrated, with engraftment rates similar to those found with other conditioning regimes, even in haploidentical HCT and associated with PTCy. In addition, the relapse rates of these RIC regimens with high doses of TMI are encouraging. On the other hand, high doses of busulfan combined with TMI may enhance the antileukemic effect of the conditioning regimen and be an option for younger patients with high-risk leukemia.

The technological gap limits the widespread use of TMI and TMLI. Technical issues in precise radiotherapy are challenging, and manual contouring of targets may take long periods (41). To increase clinical experience and the number of clinical trials with this strategy, it is necessary to develop uniform planning and treatment guidelines in radiation oncology departments and a collaborative effort between radiation oncology and the hematology team. In addition to that, the optimum chemotherapy association for each population still needs to be defined, and comparative trials with other conditioning regimen strategies are needed (42).

## Conclusion

In conclusion, TBI has been widely used in HCT at the expense of more significant toxicities. TMI or TMLI addresses the need to reduce the toxicity of TBI to critical organs while increasing the dose to selected targets through technical optimization. This strategy has been used safely and effectively in autologous HCT for multiple myeloma and allogeneic HCT for hematologic malignancies at escalating doses in combination with different chemotherapy regimen protocols. The main benefits of this technique are the possibility of intensifying the conditioning regimen, which leads to a reduced chance of relapse, without increasing transplant toxicity and transplant-related mortality. The main limitations are the availability and training of staff in this technique, long periods for manual contouring of targets, and lack of randomized studies compared with other conditioning regimens. In clinical practice, this technique can be used in association with non-myeloablative conditioning regimens in transplants of unfit or elderly patients due to its low toxicity, reducing relapse rates, including haploidentical transplantation, and also in association with myeloablative conditioning in transplants of young patients with high-risk hematological malignancies.

## Author contributions

MK and LA did the review structure, literature search, data discussion, tables, and manuscript preparation. SF and AR performed the summary of radiotherapy findings. NH participated in the design of this study and performed critical review of the manuscript. All authors provided critical review of the manuscript and approved the submitted version.

## References

[1] Gyurkocza B Sandmaier BM. Conditioning regimens for hematopoietic cell transplantation: one size does not fit all. Blood. (2014) 124:344–53. 10.1182/blood-2014-02-514778 24914142 PMC4102707

[2] Clift RA Buckner CD Appelbaum FR Sullivan KM Storb R Thomas ED. Long-term follow-up of a randomized trial of two irradiation regimens for patients receiving allogeneic marrow transplants during first remission of acute myeloid leukemia. Blood. (1998) 92:1455–6. 10.1182/blood.V92.4.1455 9694737

[3] Peters LJ Withers HR Cundiff JH Dicke KA. Radiobiological considerations in the use of total-body irradiation for bone-marrow transplantation. Radiology. (1979) 131:243–7. 10.1148/131.1.243 370902

[4] Shank B O'Reilly RJ Cunningham I Kernan N Yaholom J Brochstein J. Total body irradiation for bone marrow transplantation: the memorial Sloan-Kettering cancer center experience. Radiotherapy Oncol. (1990) 18:68–81. 10.1016/0167-8140(90)90180-5 2247651

[5] Wong JYC Liu A Han C Dandapani S Schultheiss T Palmer J. Total marrow irradiation (TMI): addressing an unmet need in hematopoietic cell transplantation—A single institution experience review. Front Oncol. (2022) 12:1003908. 10.3389/fonc.2022.1003908 36263219 PMC9574324

[6] Ali N Sharma AA de Rezende ACP. Targeted marrow irradiation intensification of reduced-intensity fludarabine/busulfan conditioning for allogeneic hematopoietic stem cell transplantation. Transplantat Cell Therapy. (2022) 28:370. 10.1016/j.jtct.2022.04.001 35421620

[7] Aydogan B Mundt AJ Roeske JC. Linac-based intensity modulated total marrow irradiation (IM-TMI). Technol Cancer Res Treat. (2006) 5:513–9. 10.1177/153303460600500508 16981794

[8] Aydogan B Yeginer M Kavak GO Fan J Radosevich JA Gwe-Ya K. Total marrow irradiation with rapidArc volumetric arc therapy. Int J Rad Oncol Biol Physics. (2011) 81:592–9. 10.1016/j.ijrobp.2010.11.035 21345619

[9] Einsele H Bamberg M Budach W. A new conditioning regimen involving total marrow irradiation, busulfan, and cyclophosphamide followed by autologous PBSCT in patients with advanced multiple myeloma. Bone Marrow Transplant. (2003) 32:593–9. 10.1038/sj.bmt.1704192 12953132

[10] Cailleteau A Maingon P Choquet S. Phase 1 study of the combination of escalated total marrow irradiation using helical tomotherapy and fixed high-dose melphalan (140 mg/m^2^) followed by autologous stem cell transplantation at first relapse in multiple myeloma. Int J Rad Oncol Biol Physics. Published online September 2022:S0360301622033946. 10.1016/j.ijrobp.2022.09.069 36174802

[11] Ladbury C Somlo G Dagis A. Long-Term follow-up of multiple myeloma patients treated with tandem autologous transplantation following melphalan and upon recovery, total marrow irradiation. Transplant Cell Therapy. (2022) 28:367–9. 10.1016/j.jtct.2022.04.022 35534000

[12] Giebel S Sobczyk-Kruszelnicka M Blamek S Saduś-Wojciechowska M Najda J Czerw T. Tandem autologous hematopoietic cell transplantation with sequential use of total marrow irradiation and high-dose melphalan in multiple myeloma. Bone Marrow Transplant. (2021) 56:1297–304. 10.1038/s41409-020-01181-x 33339899

[13] Patel P Oh AL Koshy M. A phase 1 trial of autologous stem cell transplantation conditioned with melphalan 200 mg/m ^2^ and total marrow irradiation (TMI) in patients with relapsed/refractory multiple myeloma. Leuk Lymphoma. (2018) 59:1666–71. 10.1080/10428194.2017.1390231 29065747

[14] Lin SC Hsieh PY Shueng PW Tien HJ Wang LY Hsieh CH. Total marrow irradiation as part of autologous stem cell transplantation for asian patients with multiple myeloma. Biomed Res Int. (2013) 2013:1–7. 10.1155/2013/321762 24089671 PMC3780584

[15] Somlo G Spielberger R Frankel P Karanes C Krishnan A Parker P. Total marrow irradiation: a new ablative regimen as part of tandem autologous stem cell transplantation for patients with multiple myeloma. Clin Cancer Res. (2011) 17:174–82. 10.1158/1078-0432.CCR-10-1912 21047977 PMC3717559

[16] Ogawa H Konishi T Najima Y. Phase I trial of myeloablative conditioning with 3-day total marrow and lymphoid irradiation for leukemia. Cancer Sci. (2022) 3:cas.15611. 10.1111/cas.15611 36221800 PMC9899623

[17] Stein AS Malki MMA Yang D Palmer JM Tsai N-C Aldoss I. Total marrow and lymphoid irradiation with post-transplantation cyclophosphamide for patients with AML in remission. Transplant Cell Therapy. (2022) 28:368. 10.1016/j.jtct.2022.03.025 35398328 PMC9253081

[18] Haraldsson A Wichert S Engström PE Lenhoff S Turkiewicz D Warsi S. Organ sparing total marrow irradiation compared to total body irradiation prior to allogeneic stem cell transplantation. Eur J Haematol. (2021) 107:393–407. 10.1111/ejh.13675 34107104

[19] Shi L Lu X Deng D Yang L Zhao H Shen J. The safety and efficacy of a novel hypo-fractionated total marrow and lymphoid irradiation before allogeneic stem cell transplantation for lymphoma and acute leukemia. Clin Translat Rad Oncol. (2021) 26:42–6. 10.1016/j.ctro.2020.11.004 33305023 PMC7708691

[20] Jensen LG Stiller T Wong JYC Palmer J Stein A Rosenthal J. Total marrow lymphoid irradiation/fludarabine/melphalan conditioning for allogeneic hematopoietic cell transplantation. Biol Blood Marrow Transplant. (2018) 24:301–7. 10.1016/j.bbmt.2017.09.019 29032268

[21] Stein A Palmer J Tsai NC. Phase I trial of total marrow and lymphoid irradiation transplantation conditioning in patients with relapsed/refractory acute leukemia. Biol Blood Marrow Transplant. (2017) 23:618–24. 10.1016/j.bbmt.2017.01.067 28087456 PMC5382014

[22] Hui S Brunstein C Takahashi Y DeFor T Holtan SG Bachanova V. Dose escalation of total marrow irradiation in high-risk patients undergoing allogeneic hematopoietic stem cell transplantation. Biol Blood Marrow Transplant. (2017) 23:1110–6. 10.1016/j.bbmt.2017.04.002 28396164 PMC5531195

[23] Patel P Aydogan B Koshy M Mahmud D Oh A Saraf SL. Combination of linear accelerator–based intensity-modulated total marrow irradiation and myeloablative fludarabine/busulfan: a phase I study. Biol Blood Marrow Transplant. (2014) 20:2034–41. 10.1016/j.bbmt.2014.09.005 25234438

[24] Rosenthal J Wong J Stein A. Phase 1/2 trial of total marrow and lymph node irradiation to augment reduced-intensity transplantation for advanced hematologic malignancies. Blood. (2011) 117:309–15. 10.1182/blood-2010-06-288357 20876852 PMC3037752

[25] Wong JYC Forman S Somlo G Rosenthal J Liu A Schultheiss T. Dose escalation of total marrow irradiation with concurrent chemotherapy in patients with advanced acute leukemia undergoing allogeneic hematopoietic cell transplantation. Int J Rad Oncol Biol Physics. (2013) 85:148–56. 10.1016/j.ijrobp.2012.03.033 22592050 PMC4312108

[26] Malki MMA Palmer J Tsai NC Mokhtari S Hui S Tsai W. Total marrow and lymphoid irradiation as conditioning in haploidentical transplant with posttransplant cyclophosphamide. Blood Advances. (2022) 6:4098–106. 10.1182/bloodadvances.2022007264 35838754 PMC9327543

[27] Sarina B Mancosu P Navarria P Bramanti S Mariotti J De Philippis C. Nonmyeloablative conditioning regimen including low-dose total marrow/lymphoid irradiation before haploidentical transplantation with post-transplantation cyclophosphamide in patients with advanced lymphoproliferative diseases. Transplant Cell Therapy. (2021) 27:492. 10.1016/j.jtct.2021.03.013 33857448

[28] Wong JYC Rosenthal J Liu A Schultheiss T Forman S Somlo G. Image-guided total-marrow irradiation using helical tomotherapy in patients with multiple myeloma and acute leukemia undergoing hematopoietic cell transplantation. Int J Rad Oncol Biol Physics. (2009) 73:273–9. 10.1016/j.ijrobp.2008.04.071 18786784 PMC3896447

[29] Köksal M Kersting L Schoroth F Garbe S Koch D Scafa D. Total marrow irradiation versus total body irradiation using intensity-modulated helical tomotherapy. J Cancer Res Clin Oncol. (2023) 6:2. 10.1007/s00432-022-04565-2 36607428 PMC10356893

[30] Saldi S Fulcheri CPL Zucchetti C Abdelhamid AMH Carotti A Pierini A. Impact of total marrow/lymphoid irradiation dose to the intestine on graft-versus-host disease in allogeneic hematopoietic stem cell transplantation for hematologic malignancies. Front Oncol. (2022) 12:1035375. 10.3389/fonc.2022.1035375 36568236 PMC9773831

[31] Kim JH Stein A Tsai N Schultheiss TE Palmer J Liu A. Extramedullary relapse following total marrow and lymphoid irradiation in patients undergoing allogeneic hematopoietic cell transplantation. Int J Rad Oncol Biol Physics. (2014) 89:75–81. 10.1016/j.ijrobp.2014.01.036 24725691

[32] Han C Liu A Wong JYC. Estimation of radiation-induced, organ-specific, secondary solid-tumor occurrence rates with total body irradiation and total marrow irradiation treatments. Pract Radiat Oncol. (2020) 10:e406–14. 10.1016/j.prro.2020.03.006 32302694

[33] Ladbury C Armenian S Bosworth A He T Wong FL Dandapani S. Risk of subsequent malignant neoplasms following hematopoietic stem cell transplantation with total body irradiation or total marrow irradiation: insights from early follow-up. Transplant Cell Therapy. (2022) 28:860. 10.1016/j.jtct.2022.09.013 36167306

[34] Siegel RL Miller KD Fuchs HE Jemal A. Cancer statistics, 2021. CA A Cancer J Clin. (2021) 71:7–33. 10.3322/caac.21654 33433946

[35] Attal M Lauwers-Cances V Hulin C. Lenalidomide, bortezomib, and dexamethasone with transplantation for myeloma. N Engl J Med. (2017) 376:1311–20. 10.1056/NEJMoa1611750 28379796 PMC6201242

[36] Richardson PG Jacobus SJ Weller EA Hassoun H Lonial S Raje NS. Triplet therapy, transplantation, and maintenance until progression in myeloma. N Engl J Med. (2022) 387:132–47. 10.1056/NEJMoa2204925 35660812 PMC10040899

[37] Moreau P. Comparison of 200 mg/m2 melphalan and 8 Gy total body irradiation plus 140 mg/m2 melphalan as conditioning regimens for peripheral blood stem cell transplantation in patients with newly diagnosed multiple myeloma: final analysis of the Intergroupe Francophone du Myelome 9502 randomized trial. Blood. (2002) 99:731–5. 10.1182/blood.V99.3.731 11806971

[38] Blade J. High-dose therapy intensification compared with continued standard chemotherapy in multiple myeloma patients responding to the initial chemotherapy: long-term results from a prospective randomized trial from the Spanish cooperative group PETHEMA. Blood. (2005) 106:3755–9. 10.1182/blood-2005-03-1301 16105975

[39] Scott BL Pasquini MC Logan BR. Myeloablative vs. reduced-intensity hematopoietic cell transplantation for acute myeloid leukemia and myelodysplastic syndromes. JCO. (2017) 35:1154–61. 10.1200/JCO.2016.70.7091 28380315 PMC5455603

[40] Clift RA Buckner CD Appelbaum FR Bearman SI Petersen FB Fisher LD. Allogeneic marrow transplantation in patients with acute myeloid leukemia in first remission: a randomized trial of two irradiation regimens. Blood. (1990) 76:1867–71. 2224134

[41] Schultheiss TE Wong J Liu A Olivera G Somlo G. Image-guided total marrow and total lymphatic irradiation using helical tomotherapy. Int J Rad Oncol Biol Physics. (2007) 67:1259–67. 10.1016/j.ijrobp.2006.10.047 17336225

[42] Wong JYC Filippi AR Scorsetti M Hui S Muren LP Mancosu P. Total marrow and total lymphoid irradiation in bone marrow transplantation for acute leukaemia. Lancet Oncol. (2020) 21:e477–87. 10.1016/S1470-2045(20)30342-9 33002443

